# A New Elastic Gum

**Published:** 1879-01

**Authors:** 


### ARTICLE VI.

### *A New Elastic Gum.*

A rival to India rubber and gutta-percha has been found in a new elastic gum which has been named Balata. This is the milky sap of the bully-tree that flourishes on the banks of the Orinoco and the Amazon in South America. The operation of winning the gum is similar in every respect to that employed with caoutchouc and gutta-percha. It resembles gutta-percha so closely in its general properties that much of it is shipped from Guiana and sold yearly for gutta-percha, although it has many points of superiority. It is tasteless, gives an agreeable odor on being warmed, may be cut like gutta-percha, is tough and leathery, is remarkably flexible and far more elastic than gutta-percha. It becomes soft, and may be joined piece to piece like gutta-percha, at about 120° Fah., but requires 270° Fah., before melting (higher than gutta-percha.) It is completely soluble in benzole and carbon disulphide in the cold. Turpentine dissolves it with the application of heat, while it is only partly soluble in anhydrous alcohol and ether. It becomes strongly electrified by friction, and is a better insulator of heat and electricity than gutta-percha, on which account it may find considerable application for electrical and telegraphical uses. Caustic alkalies and concentrated hydrochloric acid do not attack it, but concentrated sulphuric and nitric acids attack it as they do gutta-percha, which it closely resembles in all other properties.—*Polytechnic Review.*

